# Measuring effectiveness in immunoglobulin A nephropathy: what are we looking for?

**DOI:** 10.1093/ckj/sfag059

**Published:** 2026-05-29

**Authors:** Jonathan Barratt, Jürgen Floege, Richard Lafayette, Heather N Reich, Vladimír Tesař, Hernán Trimarchi, Shikha Wadhwani

**Affiliations:** Department of Cardiovascular Sciences, College of Life Sciences, University of Leicester, Leicester, UK; Department of Nephrology and Clinical Immunology, Rheinisch-Westfälische Technische Hochschule Aachen University Hospital, Aachen, Germany; Division of Nephrology, Department of Medicine, Stanford University, Stanford, CA, USA; Division of Nephrology, University Health Network, Department of Medicine, University of Toronto, Toronto, ON, Canada; Department of Nephrology, First Faculty of Medicine and General University Hospital, Charles University, Prague, Czech Republic; Nephrology Service, Hospital Británico de Buenos Aires, Buenos Aires, Argentina; Division of Nephrology, Department of Medicine, The University of Texas Medical Branch, Galveston, TX, USA

## INTRODUCTION

The recognition that immunoglobulin A nephropathy (IgAN) frequently leads to kidney failure in both adults and children has increased globally in recent years, based on population-level analyses of outcomes in patients receiving optimized supportive treatment as standard of care (SoC) [1]. These studies have highlighted the need for disease-modifying therapies that slow the progression of IgAN. New therapies have been shown to reduce the rate of loss of kidney function in Phase 3 studies, and many others are in development [1–4]. The challenge is determining how these new treatments fit into a comprehensive future management strategy for patients with IgAN, bringing this immune complex (IC)-mediated glomerular disease (GD) under immunologic control and preventing both IgAN-induced and generic drivers of nephron loss [1]. A key aspect of any strategy will be the ability to precisely measure the effectiveness of each intervention. Here we review the current position on measuring treatment effectiveness in IgAN.

## PROTEINURIA AND EGFR

Persistent proteinuria is a well-established surrogate marker for kidney outcomes in IgAN, being strongly associated with worsening kidney function [1, 5, 6]. Sustained proteinuria reduction (toward healthy levels) is thus a key therapeutic goal, and monitoring proteinuria helps assess disease activity and guide treatment decisions. In clinical trials, proteinuria is typically assessed using 24-hour urine samples; in clinical practice, however, errors are easily made when collecting samples. Overnight, first- or second-void, or random sample collections are often used to estimate proteinuria [7]. However, spot urine tests, although more convenient for patients and clinicians, are susceptible to single-point variability (level of hydration, time of day, diet, etc.). Compared with proteinuria, albuminuria has not yet been carefully evaluated as a modifiable risk factor in IgAN. It behaves similarly to proteinuria [8, 9], but the exact ranges in terms of risks and targets have not been carefully assessed. Further research is therefore needed.

Estimated glomerular filtration rate (eGFR) is a direct measure of kidney function, and reduction in eGFR is a major risk factor for further disease progression. As IgAN is progressive and kidney damage is irreversible (i.e. “time equals nephrons”), it is vital to monitor disease course and treatment response accurately and from diagnosis, to help avoid kidney failure. Two-year eGFR change was utilized as a surrogate marker for approval of nefecon (NefIgArd) and sparsentan (PROTECT) in IgAN [2, 3]. As eGFR values are derived and can also be highly variable, modeling is often used to best characterize eGFR changes over time [2, 3]. A 2022 analysis of published interventional studies reported that an eGFR slope over only 1 year was a significant independent predictor of treatment effects on “hard” endpoints (major eGFR loss, kidney failure) [5]. While there was also a relationship (albeit less robust) between treatment effects on both 1-year proteinuria reduction and those same hard endpoints, this did not appear to be independent of the 1-year eGFR slope [5]. It should be noted that measuring short-term changes in eGFR slope may not be as applicable in real-world practice as in clinical trials for monitoring disease progression and treatment effectiveness (because of intra-patient variability in serum creatinine or cystatin C measurements, etc.). While graphical depiction of eGFR and proteinuria as well as an automated calculation of annual eGFR loss may be of benefit to clinicians, future implementation research should focus on how best to integrate these measures into therapeutic decision-making. However, these findings suggest that, while proteinuria reduction remains an important treatment goal, efforts should be made to use short-term changes in both proteinuria and eGFR to predict outcomes and assess treatment effectiveness as early as possible [10].

It is worth noting that the baseline level and reduction of proteinuria both affect future eGFR protection, but that the protection may also be different for therapies that target the immunologic basis of IgAN compared with drugs with a predominantly hemodynamic effect [2, 3]. These emerging data suggest that when predicting future eGFR benefit, *how* proteinuria is reduced may be more important than *by what amount*. However, reducing proteinuria (to <0.5 g/day or ideally <0.3 g/day) remains an important treatment goal, as reflected in the latest Kidney Disease: Improving Global Outcomes guidelines [10].

## NON-VISIBLE HEMATURIA

While in clinical practice non-visible hematuria (when accompanied by proteinuria) is considered by some as a sign of poor prognosis in IgAN, there is a lack of evidence from prospective controlled studies or association studies that non-visible hematuria by itself is a risk factor for disease progression. This could be partly due to lack of standardized assessment methods, and logistical challenges presented by the need to use fresh urine samples in large-scale global trials. Nefecon, iptacopan, and sibeprenlimab treatment were associated with a reduction in hematuria [2, 4, 11]. However, in the NefIgArd, APPLAUSE-IgAN, and VISIONARY trials, only ∼70% to ∼75% of patients had hematuria at baseline. In addition, in patients treated with placebo, the proportion of patients without hematuria increased by seven percentage points in NefIgArd, and hematuria was no longer present in 16% of patients with hematuria at baseline in APPLAUSE-IgAN (the proportion of patients with hematuria in the placebo group of the VISIONARY study was only slightly lower after 48 weeks versus baseline) [2, 4, 11]. Therefore, there is little evidence to support using non-visible hematuria by itself as a measure of treatment effectiveness [4]. However, hematuria is the most common clinical feature of IgAN, and persistent hematuria has been reported to correlate significantly with mesangial hypercellularity in biopsy samples [12], suggesting it is a biomarker for disease activity. It is possible that non-visible hematuria is a marker of transient IgAN activity; as a result, it could provide insights into real-time disease control. Notably, standardization of the way in which non-visible hematuria is evaluated will be needed in order to validate its usefulness as a biomarker.

## IMMUNOLOGIC BIOMARKERS

The gut mucosal surface is considered to be a major source of galactose-deficient IgA1 (Gd-IgA1) and, according to the “4-hit” hypothesis, Gd-IgA1 overproduction in mucosal tissue results in its recognition by circulating anti–Gd-IgA1 antibodies and formation of Gd-IgA1–containing ICs [1]. These are deposited in the glomerular mesangium and lead to complement activation, mesangial proliferation and sclerosis, interstitial inflammation, and tubular atrophy [1]. Higher serum levels of Gd-IgA1 and anti–Gd-IgA1 antibodies are associated with IgAN progression and worse kidney outcomes [1]. Increased levels of circulating Gd-IgA1 are also associated with increased production of B-cell survival factors BAFF and APRIL [1].

Of agents currently approved for use in IgAN, nefecon has the most extensively studied immunologic biomarker profile to date. Data from the Phase 2 NEFIGAN and Phase 3 NefIgArd trials reported rapid and sustained decreases in both Gd-IgA1 and pathogenic IgA/IgG ICs with nefecon versus SoC (without significant decreases in total IgG and IgA) [13, 14]. Nefecon was also associated with decreased circulating levels of BAFF versus SoC alone. Levels of lymphocyte activation markers [cluster of differentiation 23 (CD23), CD27, and CD30] and of several chemokines [C-X-C motif chemokine 5 (CXCL5), CXCL13, and chemokine (C-C motif) ligand 19 (CCL19)] were also reduced [13]. These findings may suggest a beneficial effect with nefecon on the underpinning IgAN pathophysiology via its action on the gut mucosa. In addition, treatment with nefecon was associated with a reduction in urine-soluble CD163/creatinine excretion, which correlated with urine excretion of dickkopf-3. These changes also correlated with a reduction in complement activation as determined through reductions in both the lectin (urine mannose-binding lectin/creatinine) and alternative (plasma mannose-binding lectin-associated serine protease 3) pathways, suggesting a treatment-related reduction in factors driving glomerular and tubulointerstitial inflammation and fibrosis [13]. Studies with agents including sparsentan, iptacopan, sibeprenlimab, and others that are still in development (e.g. atacicept) have also reported positive effects on a range of immunologic biomarkers.

While these findings are indeed promising, further studies are needed to elucidate which biomarkers are most closely related to histologic changes in the kidney (e.g. via repeat kidney biopsy). All biomarkers also need validation as indicators of treatment effectiveness and standardization for routine testing and monitoring procedures.

## PROs

In recent years, there have been efforts to characterize patient-reported outcomes (PROs) in IgAN clinical trials. The NefIgArd and PROTECT trials assessed health-related quality of life (HRQoL) using 36-item Short Form Survey (SF-36) and The Kidney Disease Quality of Life–36 (KDQOL-36), respectively; both trials reported that neither nefecon nor sparsentan worsened patient HRQoL versus SoC or active control [15, 16]. To date, however, there is no validated clinical outcome assessment instrument for patients with IgAN or other GDs, and neither SF-36 nor KDQOL-36 are designed specifically for GD [17].

The CureGN cohort utilized the PROMIS scoring system to assess PROs in their longitudinal GD cohort (including >600 patients with IgAN or IgA vasculitis) and found that patient-reported edema had the strongest association with each domain and had a consistent negative association with HRQoL [17]. The SONG-GD initiative developed a core outcome set across GDs and has identified several PROs prioritized by patients [18]. While kidney function, mortality, and need for dialysis/transplant were top priorities among patients with GDs, life participation/ability to work, fatigue, anxiety, and infection/immunity were also highly rated.

Given heterogeneity in both IgAN disease manifestations and time to diagnosis, further work is needed to better understand disease-related symptoms, the degree to which they affect patient HRQoL, and how PROs can contribute to an understanding of a treatment’s effectiveness.

## CONCLUSIONS

We are experiencing a transformation in management and expectations of how we should approach the treatment of IgAN. Managing downstream consequences of IgA IC–mediated kidney damage alone is not good enough for our patients. Genome-wide association studies have identified more high-risk loci in East Asian populations than in Europeans and higher incidence of more aggressive phenotypes in Asian populations. A high polygenic score for IgAN is associated with earlier onset of kidney failure, suggesting that genetic testing could aid in early risk detection. With major advances in new disease-modifying treatments being evaluated in IgAN over the past 6 years, both clinicians and patients should expect treatments that block fundamental drivers of kidney failure in IgAN. Ideally, we should aim to return the rate of loss of eGFR to the normal physiological state for all patients for the remainder of their lives, to give them the best chance of avoiding kidney failure. For most patients, the only way we are likely to achieve this is by using drugs that reduce the formation of pathogenic IgA ICs.

There is still an urgent need for discovery and validation of IgAN-specific biomarkers beyond proteinuria, to enable personalization of treatment and to monitor effectiveness of IgAN disease-modifying drugs. Investigations into the role of Gd-IgA1 and/or IC–Gd-IgA1–IgG kinetics, as well as inflammatory and fibrotic biomarkers in blood, urine, and tissue are ongoing, although none of these biomarkers have been validated at the time of publication. Assessing the impact of treatments on these markers will allow clinicians to tailor the right drug, or drug combinations, to the right patient at the right time in the natural history of the disease, particularly in high-risk patients and those starting new treatments. There is an equal need to better understand the impact of living with IgAN on patient HRQoL, and for tools to reliably monitor HRQoL as a means of assessing treatment effectiveness. Utilizing markers such as eGFR, proteinuria, and biomarker panels, plus tools to determine disease progression and HRQoL that are implementable at scale, is vital. The global burden of IgAN also necessitates the use of broadly adoptable monitoring approaches.

To appreciate what a drug will deliver for our patients, we need measures of disease-specific modification; in particular, IgA IC formation and mesangial deposition, glomerular inflammation, and fibrosis mediators. We are making progress by analyzing bio-repositories from Phase 2 and 3 clinical trials. We now need to see these findings integrated with clinical data into validated clinical treatment algorithms to help us decide how to use or combine new treatments to prevent kidney failure in all patients with IgAN.
